# Somatic mtDNA Mutation Spectra in the Aging Human Putamen

**DOI:** 10.1371/journal.pgen.1003990

**Published:** 2013-12-05

**Authors:** Siôn L. Williams, Deborah C. Mash, Stephan Züchner, Carlos T. Moraes

**Affiliations:** 1 Department of Neurology, University of Miami, Miller School of Medicine, Miami, Florida, United States of America; 2 Department of Molecular and Cellular Pharmacology, University of Miami, Miller School of Medicine, Miami, Florida, United States of America; 3 Dr. John T. Macdonald Foundation Department of Human Genetics, University of Miami, Miller School of Medicine, Miami, Florida, United States of America; 4 John P. Hussman Institute for Human Genomics, University of Miami, Miller School of Medicine, Miami, Florida, United States of America; 5 Department of Cell Biology and Anatomy, University of Miami, Miller School of Medicine, Miami, Florida, United States of America; University of Pittsburgh, United States of America

## Abstract

The accumulation of heteroplasmic mitochondrial DNA (mtDNA) deletions and single nucleotide variants (SNVs) is a well-accepted facet of the biology of aging, yet comprehensive mutation spectra have not been described. To address this, we have used next generation sequencing of mtDNA-enriched libraries (Mito-Seq) to investigate mtDNA mutation spectra of putamen from young and aged donors. Frequencies of the “common” deletion and other “major arc” deletions were significantly increased in the aged cohort with the fold increase in the frequency of the common deletion exceeding that of major arc deletions. SNVs also increased with age with the highest rate of accumulation in the non-coding control region which contains elements necessary for translation and replication. Examination of predicted amino acid changes revealed a skew towards pathogenic SNVs in the coding region driven by mutation bias. Levels of the pathogenic m.3243A>G tRNA mutation were also found to increase with age. Novel multimeric tandem duplications that resemble murine control region multimers and yeast ρ^−^ mtDNAs, were identified in both young and aged specimens. Clonal ∼50 bp deletions in the control region were found at high frequencies in aged specimens. Our results reveal the complex manner in which the mitochondrial genome alters with age and provides a foundation for studies of other tissues and disease states.

## Author Summary

Mitochondria are unique among animal organelles in that they contain their own multi-copy genome (mtDNA). For the past 20 years it has been known that tissues like brain and muscle accumulate somatic mtDNA mutations with age. Because individual mtDNA mutations are present at very low levels, few details are known about the spectrum of mutations associated with aging. Advances in sequencing technology now permit the examination of mtDNA mutations at high resolution. We have examined the spectrum of mtDNA mutations present in putamen, a brain region prone to the accumulation of somatic mtDNA mutations. We were able to quantify the accumulation of clonal and non-clonal deletions in the mtDNA coding region which are known to have a strong association with aging. Partial deletions and novel duplications of the mtDNA control region were also identified, and appear to be more prevalent than previously recognized, but levels showed weaker associations with age than coding region deletions. Single nucleotide variants accumulate fastest in the control region, with a skew towards the accumulation of pathogenic mutations in the coding region. Understanding how the mitochondrial genome alters with age provides a benchmark for studies of somatic mtDNA mutations and dissection of the role they play in normal aging and degenerative diseases.

## Introduction

The accumulation of heteroplasmic mitochondrial DNA (mtDNA) mutations is a well-accepted facet of the biology of aging [1]. Heteroplasmic single nucleotide variants (SNVs) which are predominately transitions have been identified in all regions of mtDNA in aged tissues. Heteroplasmic deletions that fall in the major arc between the origins of replication also accumulate with age. These so-called “major arc” deletions are generally associated with pairs of direct repeats that flank the deleted region [2]. At the tissue level major arc deletions tend to be heterogeneous and of low clonality. A key exception is the clonal “common” deletion [3], that occurs between two 13 bp direct repeats and is tightly associated with aging in brain [4]. mtDNA deletions accumulate to higher levels in post-mitotic tissues such as brain, heart and muscle [5]. Within brain the distribution of somatic deletions [6] appears to correlate with regional differences in mitochondrial oxidative phosphorylation activity [7]. At the cellular level, somatic mtDNA mutations accumulate stochastically to very high levels in a minority of cells [8], [9] through clonal expansion [10] of both *de novo* and inherited variants [11], [12]. These mechanisms dictate that specific mtDNA variants are present at very low levels within a tissue [13]. As a result, most of our understanding of somatic mtDNA mutation has come from the investigation of single mutations or single classes of mutation. To provide a more comprehensive picture of somatic changes to mtDNA, we have used next generation sequencing (NGS) of mtDNA-enriched DNA (Mito-Seq) to investigate mtDNA from putamen of young and aged donors at high coverage (Sample details provided in Table S1).

## Results and Discussion

### Coding Region Rearrangements

**Figure 1:**
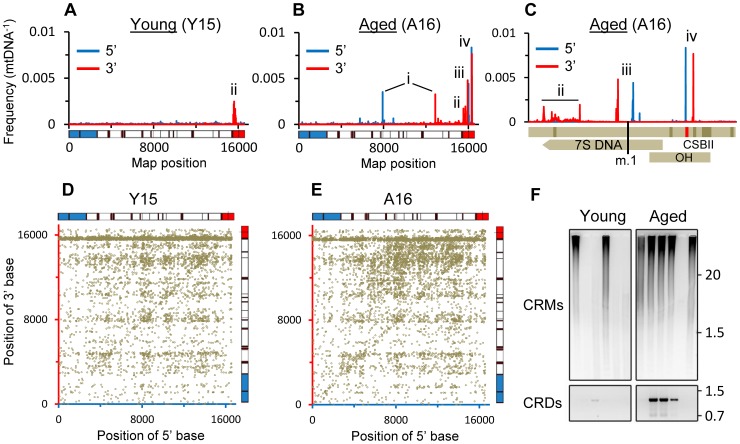
Characteristic landscape of mtDNA rearrangements in putamen. (A–C) 5′ and 3′ breakpoint position frequencies from representative young (A) and aged (B) specimens, and (C) detail of control region from (B). mtDNA map below (A) and (B) depicts rRNA genes (blue), tRNA genes (black bars), protein coding genes (white) and the control region (red). Map uses alternate numbering with a contiguous control region and m.1 as the 5′ base of *MT-TF*. In the map below (C), the top bar depicts the control region (light shading) with features indicated left to right (dark shading): termination associated sequence; conserved sequence boxes I, II (CSBII (red)), and III; light-strand promoter and heavy strand promoter-1. Middle bar shows the 7S DNA with an arrow at the 3′ end and lower bar marks heavy strand origin of replication (OH). The first base of conventional numbering is indicated (m.1). Where present (A–C), i = common deletion, ii = 3′ clustered breakpoints, iii = CRMs, iv = CRDs. (D) Dot-plot of mtDNA breakpoint distribution from (A) and (E) breakpoint distribution from (B) with axes colored accordingly and data normalized for coverage. Equivalent data to that in panels A–E for all samples is presented in Figs. S1, S3 & S4. (F) Upper panel, resolution of large amplicons from PCR of CRMs using inverted primers; Lower panel, PCR of CRDs using a breakpoint-specific primer. Sample order for both panels as in Table S1. Molecular weight markers indicated (Kb).

**Figure 2:**
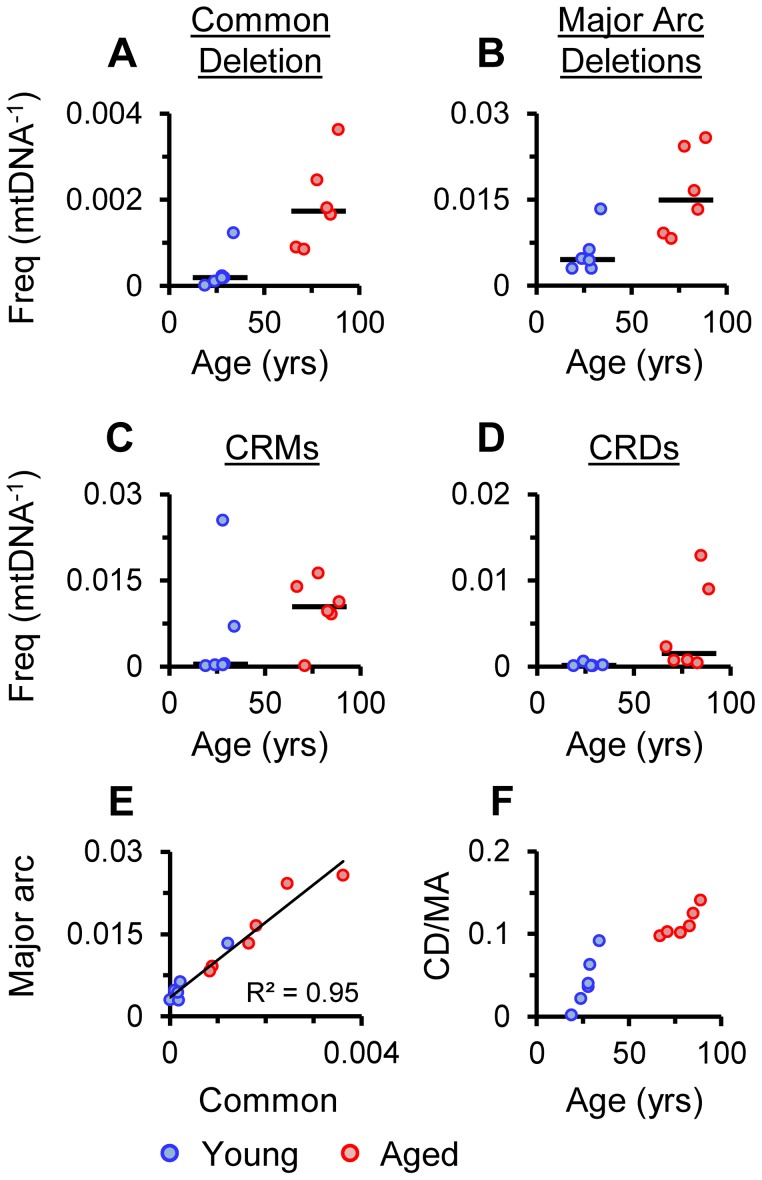
Frequencies of specific re-arrangements in putamen mtDNA. (A) Frequency of the common deletion per mtDNA (mtDNA^−1^), (B) Major-arc deletions, (C) CRMs, (D) CRDs. Bars indicate cohort medians. (E) Frequency of the common deletion (Common) versus major arc deletions excluding the common deletion (Major arc), linear regression shown with R^2^. (F) Changes in the levels of the common deletion as a proportion of cumulative major arc deletions excluding the common deletion (CD/MA) relative to age.

Breakpoints indicative of mtDNA rearrangements such as deletions, were detected by BLAST alignment. The common deletion, m.8483_13459del4977, was easily identifiable as a pair of clonal breakpoints in the coding region of aged samples (Figs. 1A–B). In agreement with other studies [6], [14], frequencies were significantly higher in the aged cohort than in the young cohort (P = 0.0087, Fig. 2A) and ranged from 8.4×10^−4^ to 3.6×10^−3^ mtDNA^−1^. The eldest specimen in the young cohort, Y12 (34 yrs), carried the deletion at 1.2×10^−3^ mtDNA^−1^ in line with observations that some individuals accumulate deletions from the third decade of life [6], [15]. Additional clonal and non-clonal deletions in the major arc between the mtDNA origins of replication are also associated with aging [16]. Dot-plots revealed “major arc” deletions as a consistent cloud of canonical breakpoints in aged specimens (Figs. 1D–E). The distribution of breakpoints in each sample matched pooled data from multiple studies of clonal deletions [17], demonstrating the extreme heterogeneity of breakpoints within individual tissue specimens. Cumulative frequencies were significantly higher in aged putamen than young (P = 0.0152) and ranged from 0.8×10^−2^ to 2.6×10^−2^ mtDNA^−1^ (Fig. 2B). As with the common deletion, Y12 carried levels of major arc deletions within the aged cohort range. Assuming a simple linear model for the accumulation of deletions, our data showed that major arc deletions accumulated faster than the common deletion (Table 1). Levels of the common deletion increased 12.5-fold and major arc deletions 3.6-fold between 25 and 80 years of age. There appeared to be a close relationship between the frequencies of the common deletion and other major arc deletions (Fig. 2E). The proportion of total major arc deletion load accounted for by the common deletion appeared to be biphasic, increasing to a plateau at about age 40 and then increasing again beyond age 80 (Fig. 2F). It is possible this pattern reflects differences in the contribution of non-clonal *de novo* deletion and clonal expansion of the common deletion, to the total mtDNA deletion load with age.

**Table 1:** Rates of accumulation and loads of mtDNA rearrangements and SNVs.

Feature	Rate (yr^−1^)	25 years	80 years
Common deletion	3.35±1.5×10^−5^	0.16±0.65×10^−3^	2.00±0.65×10^−3^
Major arc deletions	22.36±12.0×10^−5^	4.71±5.03×10^−3^	17.01±5.03×10^−3^
All SNVs^a^	6.67±2.9×10^−3^	0.23±0.12	0.60±0.12
Transitions^a^	3.69±0.7×10^−3^	0.15±0.03	0.36±0.03
All SNVs^a^ (bp^−1^)	4.02±1.8×10^−7^	1.38±0.70×10^−5^	3.59±0.70×10^−5^
Transitions^a^ (bp^−1^)	2.22±0.4×10^−7^	0.93±0.16×10^−5^	2.15±0.16×10^−5^

Rates of accumulation of mtDNA deletions and SNVs assuming linear models of accumulation (±95% CI). Frequencies per mtDNA (mtDNA^−1^) at 25 and 80 years of age determined from these rates (±RMSE).

a For SNVs, values at 25 and 80 years of age are calculated from corresponding rates applied to a baseline SNV load at 1 year determined by Kennedy *et al* [52].

### Control Region Rearrangements

Two novel rearrangements were detected in the control region. The first, m.(16508_16544)_(16565_57)dup (Fig. 1C) was present at up to 2.5×10^−2^ mtDNA^−1^, a similar range to that of major arc deletions, although there was not a significant difference in frequency between cohorts nor association between frequency and age (Fig. 2C). These breakpoints resemble mtDNA control region multimers (CRMs) we previously identified in brain and heart of the progeroid *Polg^D257A/D257A^* mtDNA mutator mouse [18]. CRMs are large species composed of multimeric tandem duplications of part of the control region with very little or no other mtDNA sequence. This sequence structure, composed of multiple short tandem repeats, is very similar to that of ρ^−^ mtDNAs in yeast [19]. We speculate that given their large size, any potential pathology associated with CRMs would likely be due to perturbation of nucleoid distribution.

In *Polg^D257A/D257A^* mice, CRM repeat units have a mean length of 566 bp and a range from ∼200–800 bp. Repeat units in human putamen were shorter with a mean length of 81 bp and ranging from 44–87 bp with the most prominent form being m.16509_22dup. Similar to CRMs in *Polg^D257A/D257A^* mice, direct repeats of 3 bp or larger were present in only 4% of CRM breakpoints suggesting they arise through a form of non-homologous end joining (NHEJ). This contrasts with major arc deletions which predominantly occur between direct repeats, inferring a role for homologous recombination [20], [21], and the present study where 83% of canonical breakpoints in the coding region involved direct repeats of 3 bp or longer. The presence of CRMs in our original putamen DNA samples was verified using inverted primer PCR and as seen in *Polg^D257A/D257A^* mice [18] this resulted in large heterogeneous amplicons (Fig. 1F). Applying the same PCR to DNA from cerebellum of the cases under study, we were unable to amplify CRM products (Fig. S2). Thus CRMs may be localized to regions that are sensitive to mitochondrial dysfunction [7] and accumulate higher levels of mtDNA damage [14]. The physiological impact of CRMs remains to be determined. Levels in putamen are ∼200-fold lower than in brain from *Polg^D257A/D257A^* mice [18] where CRMs were associated with a 45% depletion in mtDNA and an increase in mtDNA-encoded mRNAs of ∼3-fold.

We also identified a cluster of clonal control region deletions (CRDs), m.(244_309)_(311_489)del, present at frequencies of up to 1.3×10^−2^ mtDNA^−1^, similar to that of major arc deletions in aged samples (Figs. 1B–C). These deletions disrupt conserved sequence block II (CSBII) involved in mtDNA replication and transcription termination [22]. Differences in the frequency of CRDs between the young and aged cohorts was significant (P = 0.0043, Fig. 2D). 90% of CRDs were 50 bp long and the most abundant form was m.307_356del50 which occurred between a pair of 9 bp direct repeats. The 5′ and 3′ flanking direct repeats and the resulting breakpoint encompass copies of an 11 bp degenerate sequence motif recently found to be over represented within 5 bp of mtDNA deletion breakpoints, including the flanking direct repeats of the common deletion [17]. The biological basis for the association of this motif with deletion breakpoints remains undetermined. The m.307_356del50 deletion has been reported as a somatic mutation in cancers [23] and at high levels in the saliva, blood and hair follicles from a healthy Chinese family where it was shown to elicit no effect on mtDNA levels in blood [24]. PCR of original DNA samples verified the presence of CRDs in putamen (Fig. 1F). Unlike CRMs, PCR of DNA from cerebellum revealed CRDs in three aged specimens, one of which did not carry the deletion in putamen (Fig. S2). These findings define CRDs as both transmissible and somatic mtDNA mutations and indicate that they are a more prevalent feature of mtDNA mutation spectra than previously recognized. We did not find any relationship between the levels of CRMs and CRDs, nor either of these species with the levels of major arc or common deletions. Both CRMs and CRDs appear to be distinct from previously reported tandem duplications in the control region [25], [26].

### Single Nucleotide Variants

To focus on somatic variation and reduce the confounding effects of inherited high frequency heteroplasmy, only SNVs with frequencies <0.01 bp^−1^ were considered for analysis (Fig. S5). Analysis of errors in NGS has revealed nucleotide incorporation errors during library synthesis create false SNV calls that cannot be screened using quality filtering [27]. In particular, frequencies of G>T and C>A transversions are erroneously increased due to the presence of endogenous and/or exogenously-generated 8-oxoguanine. While inherent error limited the accuracy of absolute quantitation, the high level of sequencing coverage attained in our study (mean coverage 126,538 Table S1) enabled examination of differences in SNV frequencies and rates of SNV accumulation.

**Figure 3:**
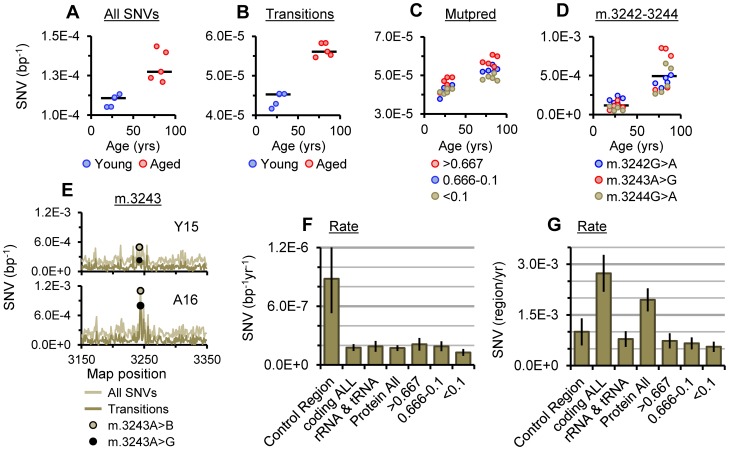
SNV burden of putamen mtDNA. (A) The average frequency of SNVs and (B) the average frequency of transitions for each sample. (C) Average SNV frequencies of transitions with Mutpred scores of >0.667, 0.666-0.1 and <0.1 (synonymous) in protein coding genes. (D) Transition frequencies between m.3242 and m.3244, bars indicate cohort medians. (E) SNV frequencies surrounding m.3243 in young and aged specimens highlighting the frequencies of m.3243A>B (A>“not A”) and the m.3243A>G transition. Plots for all samples are given in Fig. S6. Rate of accumulation per bp (F) and per mtDNA (G) for transitions in different mtDNA regions, gene classes and at protein coding bases with Mutpred scores of >0.667, 0.666-0.1 and <0.1. Error bars show ±95% CI.

In line with the consensus in the field (reviewed in [1] and [5]), the average frequency of total SNVs called in each alignment was significantly higher in mtDNA from aged putamen than young (P = 0.0079; Fig. 3A). Assuming simple linear models for SNV accumulation, we determined rates of accumulation for SNVs (Table 1). Total SNVs accumulated at 4.02±1.81×10^−7^ per base pair per year (bp^−1^yr^−1^; ±95% CI), corresponding to an increase of 2.6-fold between 25 and 80 years of age when adjusted to a baseline SNV load of 0.37±0.09×10^−5^ at less than one year of age determined for human forebrain [28]. Transitions account for about 90% of heteroplasmic SNVs [9] and are subject to significantly lower levels of NGS library-error than transversions [27]. Correspondingly, data for transitions were tighter than for total SNVs (Fig. 3B), providing a much more accurate picture of the SNV spectrum. In our putamen samples, transitions accumulated at a rate of 2.22±0.42×10^−7^ bp^−1^yr^−1^ across the entire mitochondrial genome, corresponding to a 2.3-fold increase from 25 to 80 years of age. These rates place the SNV loads for human putamen at 80 years of age (Table 1) in good agreement with published values for aged forebrain [27], [28] and human colonic crypts [9] which range from ∼2.2–3.5×10^−5^ bp^−1^.

Both of the above somatic SNV mutation rates are an order of magnitude higher than mutation rates for germline mtDNA haplotypes calculated from phylogenic studies [29], [30], likely reflecting the influence of purifying selection on the fixation of germline variants [31]. As seen in phylogenic [29], [30] and pedigree studies [32] of germline mtDNA, and in somatic SNV analysis [27] the most abundant SNVs clustered in the control region in both young and aged samples (data not shown). Although the average frequency of control region transitions remained significantly higher in the aged cohort than the young (P = 0.0079). A plausible explanation for the clustering of SNVs in the control region is that a significant proportion of variance in this region is inherited. In addition, given the role of the control region in mtDNA replication and maintenance [33], expansion of variant mtDNA clones may drive increased somatic variance in this region as opposed to *de novo* mutation. Alternatively, as the control region is the most variable region of mtDNA [34] and this may simply reflect tolerance of sequence variation in this region. The rate of accumulation of transitions in the control region was 5-fold higher than the rate in the coding region (8.82±3.5×10^−7^ bp^−1^yr^−1^ and 1.77±0.4×10^−7^ bp^−1^yr^−1^ respectively, Fig. 3F). Again both values are an order of magnitude higher than germline mutation rates for these regions calculated from phylogenic data [29], [30]. However, in alignment with the determination of more rapid substitution rates when calculated over shorter timescales [35], they are very close to germline mutation rates calculated from pedigree analysis by Howell and coworkers [32]. In this study analysis of blood from multi-generational pedigrees combined with information from similar studies revealed mutation rates of 9.5×10^−7^ bp^−1^yr^−1^ in the control region and 1.5×10^−7^ bp^−1^yr^−1^ in the coding region (5.3–15.7×10^−7^ bp^−1^yr^−1^ and 0.2–4.9×10^−7^ bp^−1^yr^−1^ respectively at 99.5% CI). While more work is necessary, this raises the intriguing possibility that apparent mtDNA SNV mutation rates may be similar in somatic and germline tissues. As there is clear evidence for purifying selection of germline mtDNA [36], which should lower the germline mutation rate, the similarity may reflect the antagonistic effect of the rapid expansion of permissive germline variants at replication bottlenecks during germ cell development [37].

When corrected for the difference in size of the coding and control regions, the rate of accumulation of mutations within each of these regions per mtDNA was 2.7-fold higher for the coding region than the control region. This demonstrates that coding region mutations still constitute the major burden of somatic variance per mtDNA despite lower rates of accumulation per base pair (Fig. 3F). Within the coding region there was no notable difference in the rates of accumulation of transitions between RNA and protein coding genes (Fig. 3G). There did not appear to be any relationship between levels of SNVs and mtDNA rearrangements that could not be accounted for by corresponding relationships to age.

### The m.3243A>G Mutation

The heteroplasmic transition m.3243A>G in the *MT-TL1* tRNA gene is likely the most prevalent pathogenic mtDNA mutation [38] and is primarily associated with MELAS and MIDD syndromes [39]. The region surrounding m.3243 is an etiologic hotspot for mutations [40] although there have been conflicting reports as to whether m.3243A>G accumulates in normal aging [41], [42]. We observed a distinct hotspot of SNV abundance spanning m.3243 in aged samples (Figs. 3D–E) with a significantly higher average frequency for SNVs through m.3242_3244 than young samples (*P* = 0.0079 for each, <0.0001 overall). In all aged samples the most abundant SNVs called at m.3242_3244 were the transitions, m.3242G>A, m.3243A>G and m.3244G>A, all of which have been associated with mitochondrial diseases [39], [43]. There is some evidence of association between detectable levels of m.3243A>G in hair follicle DNA and age related hearing loss [38], implying this finding may have consequences for the biology of aging.

### Strand Bias in Transitions

**Figure 4:**
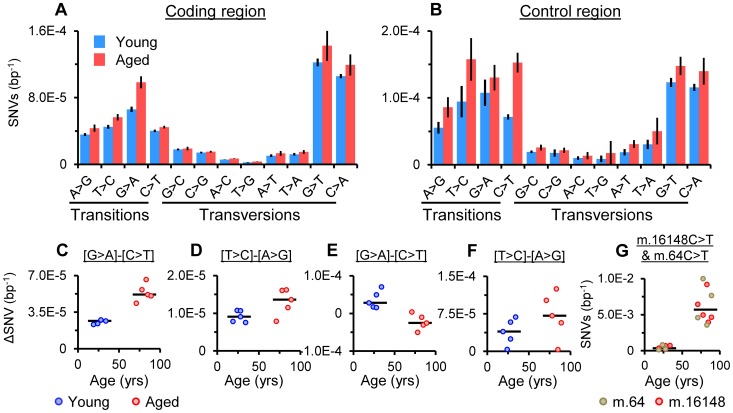
Strand bias for transitions. (A) Average frequencies for each base change in the coding region and (B) the control region of the young and aged cohorts, (± SD). (C) Magnitude of [G>A]-[C>T] bias (ΔSNV bp^−1^, the difference between G>A and C>T frequencies) and (D) [T>C]-[A>G] bias in the coding region. (E) Magnitude of [G>A]-[C>T] bias and (F) [T>C]-[A>G] bias in the control region. (G) Frequency of m.64C>T and m.16148C>T. Bars indicate cohort medians.

Applying duplex sequencing to mtDNA from human forebrain, Kennedy and coworkers have recently described a novel strand bias for somatic transitions in the mtDNA coding region, detected as increased G>A versus C>T and T>C versus A>G transitions in the reference strand (L-strand) [28]. The G>A versus C>T mutation bias is proposed to be caused by cytosine deamination (C>U) on the H-strand, potentially occurring during replication while the H-strand is exposed. As the mtDNA reference strand is the opposing L-strand the bias is manifest as an increase in the frequencies of G>A relative to C>T transitions. Dissection of SNV spectra replicated this finding in our putamen specimens. Significant differences in the frequencies of both G>A versus C>T, and T>C versus A>G transitions were observed in the coding region of young and aged samples (P = 0.0079 for each, Fig. 4A). The median bias in the G>A and C>T frequencies ([G>A]-[C>T]) in the coding region was 2.56×10^−5^ bp^−1^ in the young cohort and 5.18×10^−5^ bp^−1^ in the aged cohort, with a significant difference between cohorts (P = 0.0079, Fig. 4C). For [T>C]-[A>G] bias in the coding region, median magnitudes were lower (Fig. 4D) and differences between cohorts were not significant (P = 0.0556).

In the control region, G>A versus C>T frequencies showed a similar difference in young samples as seen in the coding region (P = 0.0079, Fig. 4B). However, in aged samples no difference in G>A versus C>T frequencies was observed. In addition, significantly lower magnitudes of [G>A]-[C>T] bias were seen within samples compared to the young cohort (P = 0.0079, Fig. 4E), indicative of an age-related switch in [G>A]-[C>T] bias, contrary to the coding region where bias appears to increase with age. Examination of SNV frequencies revealed troughs in [G>A]-[C>T] bias in the control region (Fig. S7) driven by the accumulation of high levels of m.64C>T and m.16148C>T in all aged samples (Fig. 4G). Exclusion of these variants from analysis did not recapitulate the positive [G>A]-[T>C] bias seen in the coding region in the aged cohort. Both variants occur as haplotype polymorphisms [30] (MitoMaster GenBank frequencies 2.6% and 1.7% respectively [44]) and m.64C>T has previously been noted in aged brain specimens [45]. Predicting either the consequence or origin of the accumulation of these variants is difficult as neither falls within a known mtDNA control element. While their accumulation may reflect the expansion of low consequence variants under a lack of mutational bias, it may also be that they represent unknown functional elements in the control region. Bias in control region T>C versus A>G frequencies shifted in the same direction as the coding region and was significant in both young and aged samples (*P* = 0.0159 and 0.0079 respectively) although differences in the magnitude of [T>C]-[A>G] bias between cohorts were not significant and the relationship with age was weak (Fig. 4F). As mentioned above, high G>T and C>A transversion frequencies, stemming from library synthesis base incorporation errors at 8-oxoguanine [27], were noted in the coding and control regions of all samples (Fig. 4A–B). Recent work has confirmed that *in vivo* there is no evidence for accumulation of G>T and C>A transversions with age in brain [28].

Analysis of “Mutpred” predicted pathogenicity scores [46] for germline mtDNA variants has demonstrated that variants with high predicted pathogenicity scores (>0.6), are selected against [31]. In contrast, we found that transitions with high pathogenicity scores had higher average frequencies than those with lower ones in both the young and aged cohorts (Fig. 3C), in agreement with studies of single cells from colonic crypts of aged donors [9]. The skew in pathogenicity most likely reflects the combination of mutational strand bias described above and skewed base distribution at different pathogenicity scores due codon composition (Fig. S8). However, the apparently localized increases in frequencies of SNVs at m.3242_3244, m.64C>T and m.16148C>T (Figs. 3D–E & 4G) suggests there may also be some modification of SNV spectra beyond strand bias. Transitions with pathogenicity scores >0.667 accounted for 37% of the increase in transition SNV burden at protein coding bases and 20% across all bases. At an SNV load of 0.15 mtDNA^−1^ at 25 years of age (Table 1) these percentages translate to pathogenic SNV burdens of 0.03–0.06 mtDNA^−1^, raising to 0.07–0.13 mtDNA^−1^ at age 80. Pathogenic mtDNA mutations have threshold mutation loads in tissues of 0.80–0.90 mtDNA^−1^ [47]. While it is uncertain whether heterogeneous mutations can have additive effects, this indicates that the steady-state pathogenic somatic mtDNA burden in normal putamen at 80 years of age is about 6–12-fold lower than that of a patient with a mitochondrial disorder. Nevertheless, as the etiology of the stoichiometric accumulation of somatic mtDNA mutations in aging is distinct from the inheritance of mtDNA mutations in patients with mitochondrial disorders [13], these estimates may still reflect a considerable stress.

### Analysis of Numts Using ρ^0^ Cell DNA

To examine the influence of nuclear mtDNA sequences (numts) [48] on our analysis we carried out identical sequencing of total DNA from human 143B.206 ρ^0^ cells that do not have mtDNA [49] and subjected the resulting “pseudo”-mtDNA alignment to identical analysis (For alignment details see Table S1). Only 20 breakpoints were called in this alignment compared to the 4,183–13,877 identified in putamen specimens (median 11,009). These 20 included a single call for the common deletion and a single call for a CRM. Given the hundreds of hits for verifiable species like the common deletion and CRMs in our mtDNA-enriched samples, we determined that numts had negligible influence on analysis of rearrangements. In turn, the identification of the common deletion and a CRM breakpoint in an ostensibly nuclear DNA sample implies these are evolutionarily persistent mutations. With respect to SNVs the influence of numts in determining control region clustering can be excluded as this was not observed in our ρ^0^ alignment (Fig. S9C). In addition, no SNVs were reported in our ρ^0^ cell alignment between m.3100 and m.3300, ruling out an influence of numts in relation to increased SNV frequencies spanning m.3243 (Fig. S9C). Interestingly an opposing skew in pathogenicity, towards higher average frequencies for SNVs with low predicted pathogenicity, was seen in the ρ^0^ alignment (Fig. S9B). This skew matches that seen in phylogenic studies of pathogenicity and higher mutation rates for 3^rd^ base positions in studies of mtDNA haplotype variation [29], [30]. As the ρ^0^ alignment represents numts, this skew is in agreement with the concept of nuclear transfer of evolutionarily stable mtDNA variants that predominantly have low pathogenicity scores [31].

The data presented above represent the steady state somatic mutation spectra of tissue specimens. They are likely the product of opposing biological forces that act to increase or decrease mutation loads and result in the maintenance of somatic mutation burdens at tolerable levels. Dissecting the contribution of specific factors such as de novo mutation or clonal expansion is not possible from this data. Considering the relatively small samples size, the similarities between the mutation spectra in each cohort underlines the consistency which the mitochondrial genome alters with age in putamen. Of note, the rearrangements identified in the control region warrant further study given their frequency and undetermined biological impact. It is hoped these data will provide useful comparative benchmarks for studies of somatic mtDNA mutation in other tissues and in disease states.

## Materials and Methods

DNA extraction and sequencing. mtDNA-enriched total DNA extraction was based on our previously described approach [18] with minor alterations. Putamen samples were obtained from neurologically normal fresh frozen specimens at the University of Miami brain Endowment Bank (Table S1). All donors were Caucasian males. 0.20–0.35 g tissue punches were rapidly thawed at room temperature in 4 ml of homogenization buffer (200 mM mannitol, 50 mM sucrose, 10 mM HEPES (pH 7.0), 1 mM ETDA) and homogenized using 30 strokes of a Teflon-glass Dounce homogenizer on ice. Crude mitochondrial fractions were harvested from homogenates by differential centrifugation at 600 g to clear debris and 9000 g to collect mitochondrial pellets. mtDNA-enriched DNA was obtained by resuspension in 1 mL extraction buffer (33 mM TRIS pH 8.3, 10 mM EDTA, 10 mM NaCl). To which SDS was added to 1% w/v and 3 mAU proteinase K solution (Qiagen) was added followed by incubation at 56°C for 4 hrs. Total nucleic acids were extracted twice using 25∶24∶1 phenol∶cholorfom∶isoamyl-alcohol (v/v/v) followed by two extractions with 24∶1 cholorfom∶isoamyl-alcohol (v/v). Nucleic acids were precipitated by ethanol/NaAc precipitation and resuspended in 55 uL 10 mM TRIS pH 8.5. RNA was then digested with 0.07 U RNAse A (Qiagen) and 300–500 ng dsDNA by Qubit (Invitrogen) analysis submitted for library synthesis. Libraries were prepared using Illumina Truseq PE V3-cBot-HS cluster kits and sequenced on the Illumina HiSeq 2000 platform at 5–8 libraries per lane with image processing using CASAVA V1.7/1.8 as 2×100 bp paired-end reads. Each sequencing run contained specimens from both young and aged cohorts with similar age distributions (Table S1).

Alignment. Bioinformatic analysis was done with Genomics Work Bench V4.7-5.5.2 (CLCBio). Reads were quality trimmed with an average post-trim read length >95 bp. Initial alignments were made against the revised Cambridge reference sequence (CRS) mtDNA reference sequence (NC_012920), using low stringency local alignment with a cutoff of 80% similarity over 50% length to collect mtDNA-like reads and reduce datasets. Aligned reads and sample-specific consensus sequences were extracted from these assemblies. Reads were then assembled back against respective sample-specific consensus sequences using high stringency local alignment with a cutoff of 90% similarity over 95% length. Reads that aligned at low stringency but not high stringency (generally <0.7% aligned reads) were collected for detection of rearrangement breakpoints. mtDNA haplotyping was done using MitoTool 1.1a [50].

Analysis of rearrangements. Breakpoints were identified using BLAST to align reads against NC_012920 with alternate “murine” numbering to provide a contiguous control region and a first base position at the start of *TRNF* (m.577). This alternate reference sequence enabled detailed examination of recombination involving the control region, in particular rearrangement spanning m.16569_1. To streamline output, a word length of 15 was used with open gap cost of 5 and extension cost 2. Data was parsed to collect reads with two segments in the same sense and collectively extending the full length of the read, neither of which was fully internal. Between 4,183–13,877 breakpoints were sequenced per alignment. The common deletion was quantified by counting: m.(8477_8483)_(13262_13452)del; the cumulative burden of major arc deletions was determined by counting m.(5576_15976)del>320 excluding the common deletion and corrected for putative chimeras by subtracting m.(15976_5576)del>320 (Fig. S1C); CRMs were quantified by counting m.(16492_59)_(16492_59)del>137; and CRDs m.(244_494)del (each described here with CRS numbering). The frequency of each type of rearrangement per mtDNA equivalent (mtDNA^−1^) was determined by normalizing to average coverage and assumes a single rearrangement per full length mtDNA. Data used for breakpoint dot-plots was corrected for coverage by reducing the volume of data plotted by the ratio of the average coverage of the alignment to the lowest coverage alignment, using cluster coordinates as a means to randomly shuffle reads. To reduce over interpretation of outliers and to provide conservative estimates of significance all tests of significance are two tailed Mann-Whitney rank tests.

Analysis of SNVs. High stringency assemblies were used for SNV detection using CLCBio quality-based SNP detection algorithm. The algorithm filters variant calls on the basis of quality scores for the central base (>Q33), the average quality of neighborhood window (radius ±5 bp, >Q30) and the presence of other mismatches or gaps (< = 2) within the window. Significance filtering, i.e. limits on coverage or absolute counts, were not applied as a very low counts are biologically valid in a genetically heterogeneous system especially when considering the sampling effect of cluster generation. To exclude reads from chimeric fragments [51], all reads in broken pairs were excluded from analysis. Taken together these parameters excluded a significant amount of sequencing data and reduce effective sequencing coverage by 25–30% for SNV detection. SNV tables recorded frequencies for all four possible alleles at each base. To focus on somatic variation and avoid confounding effects of inherited high frequency heteroplasmy which is common, only SNVs with frequencies <0.01 bp^−1^ were considered for analysis. Data from two sequencing runs were normalized by correcting linearly for the difference between mean SNV frequency of each run (Fig. S6). At the levels of coverage attained, the highly consistent nature of NGS sequencing error enabled detailed analysis of relative SNV frequencies but over-estimated absolute SNV levels due to incorporation errors. For examination of different classes of SNV frequencies, data was normalized by correcting linearly for the difference between means for each specific base change within the coding region across all samples between runs. These tables were used for calculation of mutation rates within different mtDNA coding and control regions, Mutpred-grouped protein coding bases and the m.3242_3244 triplet. For analysis of coding regions, SNV data from all alignments was put in phase by aligning to m.577G, there were no insertions/deletions in the coding regions of any consensus sequence. Control region length varied from −1 to +5 bp of CRS. Mutpred data tables for transitions in the CRS were taken from Pereira *et al* [31]. Fourteen codons in *MT-ATP6* where bases overlap *MT-ATP8* were considered only for *MT-ATP8*. Predicted pathogenicity scores for transitions were split into three groups, those with scores of >0.667 and non-sense mutations (3468 bp), those with scores of 0.666-0.100 (3465 bp) and synonymous mutations plus the small number of bases with scores of <0.100 (4386 bp). Transition frequencies for each base position were determined against sample-specific consensus sequences and aligned with mutpred scores calculated from CRS sequence for each base. The maximum sequence divergence between sample coding region sequence and CRS coding region sequence was 28 bp out of 15,447 bp. To reduce over interpretation of outliers and to provide conservative estimates of significance all tests of significance are two tailed Mann-Whitney rank tests.

PCR. For detection of CRMs, m.(16508_16544)_(16565_57)dup, inverted primer PCR was carried out using Kapa HiFi 2× master mix (Kapa Biosciences) containing a proofreading polymerase under manufacturers standard reaction conditions with a Tm of 62.5°C and 60 s extension time.

CRM Primers: 

 16562-F: TCACGATGGATCACAGGTCTAT

 16540-B GTGGGCTATTTAGGCTTTATGACC

For detection of the m.307_356del50 CRD we used touchdown PCR with standard non-proofreading Taq polymerase (Bioline), reaction buffer (Bioline Mango) and conditions with Tm dropping 68-61°C over the first 10 cycles, followed by another 30 cycles at a Tm of 61°C and an extension time 90 s throughout.

CRD Primers:

 CRD1-F: AAAAATTTCCACCAAACCCCAAAA

 CRD2-F: AAAAATTTTCACCAAACCCCAAAA

 1421-B CACCTTCGACCCTTAAGTTTCATA.

CRD forward primers span the m.307_356del50 breakpoint. CRD2-F contains the m.295C>T polymorphism and was used for the Haplogroup J samples (Y03 and A19, Table S1).

## Supporting Information

Figure S1 mtDNA from aged putamen displays characteristic distribution of re-arrangement breakpoints. (A) Dot-plots of breakpoints for all samples as in Figures 1D–E. Sample order arranged by increasing age left to right, top to bottom. Young cohort blue axes, aged cohort red axes, sample IDs indicated. (B) Identity dot-plot of human mtDNA (NC_012920) with white regions having <34% identity in a 200 bp window. Note the symmetrical pattern of horizontal and vertical regions of similarity closely matches the symmetrical checkerboard patterns seen in all putamen samples. (C) Dot-plot highlighting features of breakpoint landscape. Light shading demarks canonical breakpoints (e.g. m.4000_12000del) and dark shading non-canonical (e.g. m.12000_4000del) breakpoints. Rectangle outlines 3′-clustered breakpoints in the control region and the triangle defines the approximate region used to quantify major-arc deletions. The position of the common deletion is marked with a red cross.
(PDF)

Figure S2 CRMs and CRDs detected in putamen are not universally present in cerebellum. Gel showing the lack of amplification of CRM products in cerebellum DNA of the cases under study using an identical PCR to that shown in Figure 1F. Sample order as in Figure 1F and Table S1. The two right-hand lanes in the panel of aged samples are positive controls: cerebellum from an additional case spiked with 1/50 dilution of A17 putamen mtDNA and a CRM positive putamen sample. Lower panel, PCR for CRDs using breakpoint-specific primer as in Figure 1F. Lane order as upper panel. No cerebellum specimen was available for A17 hence there is a blank space in the lower panel and empty lane directly above in upper panel.
(TIF)

Figure S3 Linear plots of 5′ and 3′ breakpoint position frequencies for all samples. Y-axis scale adjusted to maximal peak heights. Map position numbering modified to fit murine map with a contiguous control region as described in Methods. Young samples in the left column and aged samples on the right, arranged by increasing age from top to bottom. mtDNA maps are given below depicting rRNA genes (blue), tRNA genes (black bars), protein coding genes (white) and the control region (red). Sample IDs are indicated.
(TIF)

Figure S4 Linear plots of 5′ and 3′ breakpoint position frequencies in the control region for all samples. Sample order and layout as in Fig. S3. Y-axis scale adjusted to visualize low frequency breakpoints truncating high frequency peaks. Control region features indicated below each column as in Fig. 1C, top bar shows entire control region (light shading) with features indicated, left to right (dark shading): termination associated sequence, conserved sequence boxes I, II (CSBII (red)) and III, Light-strand promoter and heavy strand promoter-1. Middle bar shows the 7S DNA with arrow indicating 3′ end. Lower bar defines heavy strand origin of replication (OH). CRS m.1, indicates first base of CRS numbering.
(TIF)

Figure S5 Reproducible differences in SNV frequencies between young and aged cohorts allowed normalization of SNV data from different sequencing runs. (A) Average frequencies of SNVs with a frequency of <0.01 bp^−1^ for each sample from the two sequencing runs that encompass all samples. Numbers above each plot cross-reference data in table (C) below. (B) Average frequencies of transitions with a frequency of <0.01 bp^−1^ for each sample. (C) Summary table of data relating to plots above. A-Y, mean frequency of aged cohort minus mean frequency of young cohort for each data set; ΔFrequency/yr, gradient of mean frequency vs. age. (D) Detail of SNV frequency vs. rank frequency (high-low) for SNVs with a frequency <0.01 for all samples. Samples A17 and Y13 (indicated) were considered outliers and were excluded from SNV analysis.
(PDF)

Figure S6 SNV clustering around m.3243 is observed in aged putamen. SNV frequencies with samples arranged left to right by increasing age, (A) young cohort, (B) aged cohort.
(TIF)

Figure S7 Overlaid C>T frequencies in for all young (A) and aged (B) samples. Peaks for m.64C>T and m.16148C>T indicated in (b). (C) Difference between mean [G>A]-[C>T] bias over 25 bp rolling average for young vs. aged samples aligned to above. Control region features indicated below each column as in Fig. 1C, top bar shows entire control region (light shading) with features indicated, left to right (dark shading): termination associated sequence, conserved sequence boxes I, II and III, Light-strand promoter and heavy strand promoter-1. Middle bar shows the 7S DNA with arrow indicating 3′ end. Lower bar defines heavy strand origin of replication (OH). CRS m.1, indicates first base of CRS numbering.
(TIF)

Figure S8 Bases counts as proportion of total bases in each pathogenicity group used for identifying pathogenicity skew. G>A and T>C mutations have the highest increase in frequency in the coding region and predominate in the group of transitions with the highest predicted pathogenicity.
(TIF)

Figure S9 SNV analysis of mtDNA alignment of total DNA extracted from 143B.206 ρ^0^ cells lacking mtDNA. (A) Demonstration of the absence of mtDNA in the ρ^0^ cells, using the same DNA sample as used for library synthesis. Left-hand panel shows 0.7% agarose gel of 4.5 µg of *Xho*I-digested total DNA from ρ^0^ cells and wild-type 143B ρ^+^ cells which contain mtDNA, stained with ethidium bromide (EtBr). *Xho*I linearizes mtDNA by cutting once at m.14955. Middle panel is an image from a Cyclone phosphoimager (Perkin Elmer) of a blot of the gel in the left-hand hybridized with a probe against m.13385_15311. Note the absence of mtDNA signal in the ρ^0^ lane. Right-hand panel is the same image enhanced confirming the absence of signal. In each, m = molecular weight marker (Kb), ρ^0^ = 143B.206 ρ^0^ cell DNA, ρ^+^ = 143B ρ^+^ cell DNA. Attempts to quantify the region in the ρ^0^ lane equivalent to the mtDNA band in the ρ^+^ lane found relative signal to be 300–725-fold lower, below the quantification limit for Southern blotting. Furthermore, no evidence of any band equivalent mtDNA was present in the ρ^0^ lane. (B) Average frequencies of transition pseudo-SNVs called in the ρ^0^ alignment at bases with Mutpred scores of >0.667, 0.666-0.1 and <0.1. Note the distribution is the opposite of that seen in Mito-Seq assembles (Fig. 3C). (C) The distribution and frequency of SNVs called using identical detection parameters as the putamen samples. mtDNA map is given above depicting rRNA genes (blue), tRNA genes (black bars), protein coding genes (white) and the control region (red). Note lack of clustering in the control region and the absence of calls between m.3100–m.3300 (arrow), encompassing m.3243.
(TIF)

Table S1 Summary of cases and alignment data. “% mtDNA reads” is the percentage of total sequencing reads that align to mtDNA. “SD as %” is the standard deviation in coverage as a percentage of average coverage. “Run” identifies which sequencing workflow each sample was part of. Input reads for ρ^0^ assembly was 47,991,218 and the mean for putamen libraries 46,881,984.
(PDF)
