# Correction: Somatic mtDNA Mutation Spectra in the Aging Human Putamen

**DOI:** 10.1371/annotation/4b800314-8d35-454d-afca-af6d0f57b5d1

**Published:** 2013-12-24

**Authors:** Siôn L. Williams, Deborah C. Mash, Stephan Züchner, Carlos T. Moraes

Reference [52] in the main reference list is a duplication of reference [28]. The reference to [52] in the footnote for Table 1 should instead be [28] “Kennedy SR, Salk JJ, Schmitt MW, Loeb LA (2013) Ultra-sensitive sequencing reveals an age-related increase in somatic mitochondrial mutations that are inconsistent with oxidative damage. PLoS Genet 9: e1003794. doi: 10.1371/journal.pgen.1003794”

