# Supplementary material for: Somatic mtDNA Mutation Spectra in the Aging Human Putamen
Source: PLoS Genet. 2013 Dec 5;9(12):e1003990. doi: 10.1371/journal.pgen.1003990 (PMC3854840; doi:10.1371/journal.pgen.1003990)
Supplement: Figure S1 — mtDNA from aged putamen displays characteristic distribution of re-arrangement breakpoints. (A) Dot-plots of breakpoints for all samples as in Figures 1D–E. Sample order arranged by increasing age left to right, top to bottom. Young cohort blue axes, aged cohort red axes, sample IDs indicated. (B) Identity dot-plot of human mtDNA (NC_012920) with white regions having <34% identity in a 200 bp window. Note the symmetrical pattern of horizontal and vertical regions of similarity closely matches the symmetrical checkerboard patterns seen in all putamen samples. (C) Dot-plot highlighting features of breakpoint landscape. Light shading demarks canonical breakpoints (e.g. m.4000_12000del) and dark shading non-canonical (e.g. m.12000_4000del) breakpoints. Rectangle outlines 3′-clustered breakpoints in the control region and the triangle defines the approximate region used to quantify major-arc deletions. The position of the common deletion is marked with a red cross. (PDF) [file pgen.1003990.s001.pdf]

Figure S1

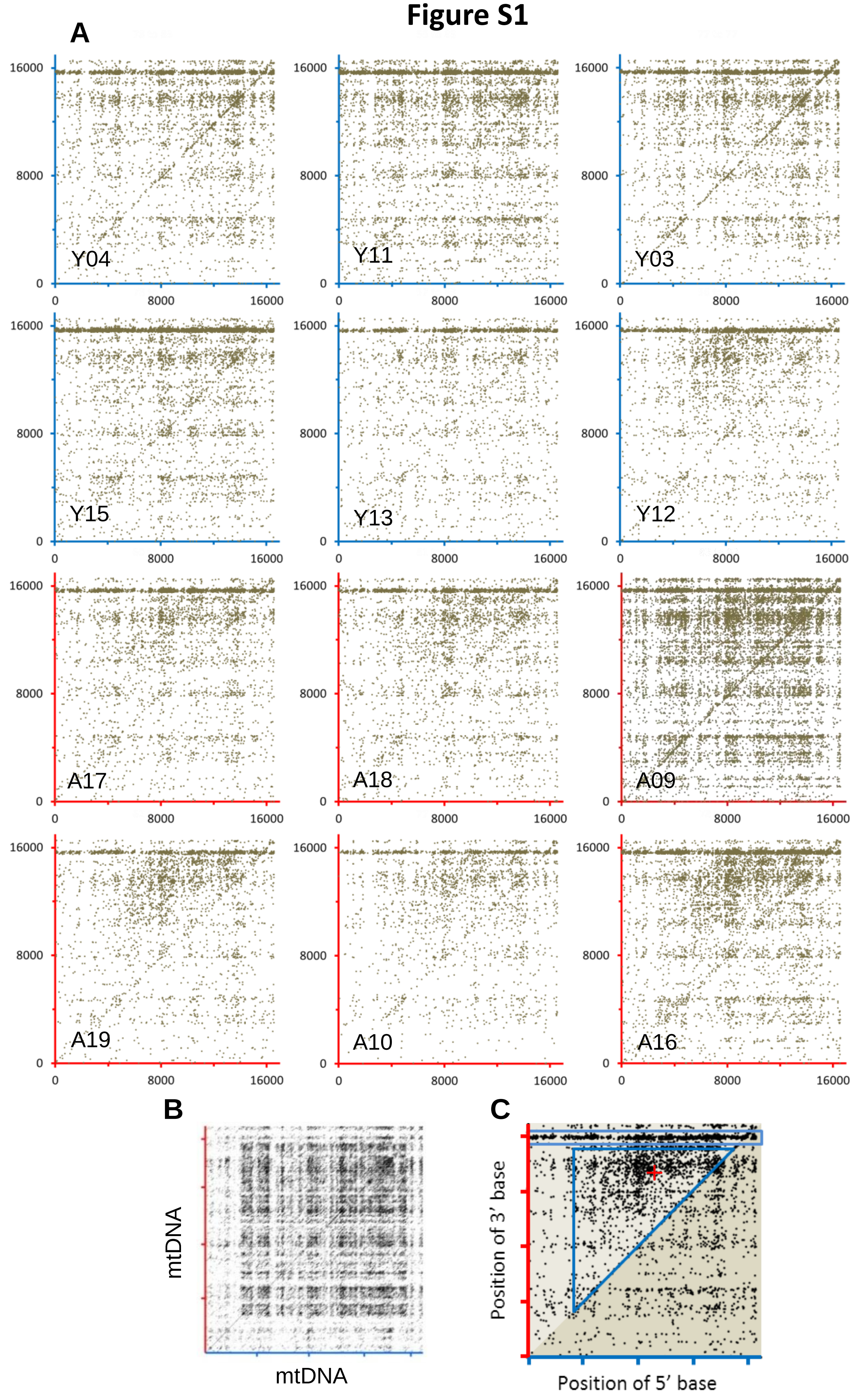
A
16000
8000
0
0
8000
16000
Y04
Y11
Y03
Y15
Y13
Y12
A17
A18
A09
A19
A10
A16
B
mtDNA
mtDNA
C
Position of 3' base
Position of 5' base

Figure S1 Commentary.

Dot-plots of breakpoints revealed a predominant “checkerboard” pattern that was symmetrical about the diagonal defining canonical and non-canonical recombination in all samples (Figs. 1D-E & S1A). We believe this patterning reflects chimeric reads generated during library preparation [51] for a number of reasons. Firstly, it is seen in all human and murine mtDNA libraries (data not shown). The pattern also overlays the distribution of sequence homology in mtDNA (Fig. S1B) and in assemblies from mice, the ratio of breakpoints in canonical versus non-canonical orientations (Fig. S1C) was extremely close to 1:1 indicating no orientation bias as would be expected in a homology-driven artifact and which is distinct from described mtDNA rearrangements [16]. Furthermore, we were never able to clone non-canonical breakpoints in the coding region using PCR-free cloning suggesting these breakpoints may not exist in pre-library DNA.

A band of 3’-clustered breakpoints in the control region was noted in all samples (Figs. 1A-E and S1A, S1C, S3). The 5’ positions of these breakpoints were distributed throughout mtDNA whereas 3’ positions were clustered between ~m.16045-m.16365 (Figs. 1C & S4). This region encompasses the 3’ end of the 7S DNA, which forms the mtDNA displacement loop (D-loop) and a prominent 3’ breakpoint in this region was m.16107, adjacent to the CTG triplet originally mapped as the 3’-end of the 7S DNA [52]. The cumulative frequency of these breakpoints had no relationship to age, ranging from 0.02-0.09 $mtDNA^{-1}$ and 0.01-0.11 $mtDNA^{-1}$ in the young and aged cohorts respectively. Short-extension long-range PCR, commonly used to identify major arc mtDNA deletions [53], failed to detect a corresponding abundance of deletions terminating in this region (data not shown). Moreover, ubiquitous mtDNA rearrangements at such high levels of abundance have never been reported. Rather than representing deletions this suggests instead that a proportion of 7S DNA molecules may have promiscuous 3’-ends with short heterogeneous extensions into other mtDNA regions. This may have implications for studies of mtDNA replication and maintenance but investigation is beyond the scope of the current study.

References for Supporting Information

52. Doda JN, Wright CT, Clayton DA (1981) Elongation of displacement-loop strands in human and mouse mitochondrial DNA is arrested near specific template sequences. Proc Natl Acad Sci U S A 78: 6116-6120.
53. Taylor RW, Wardell TM, Blakely EL, Borthwick GM, Brierley EJ, et al. (2000) Analysis of mitochondrial DNA mutations : deletions. Methods Mol Med 38: 245-264.
