# Supplementary figures and images for: Somatic mtDNA Mutation Spectra in the Aging Human Putamen
Source: PLoS Genet. 2013 Dec 5;9(12):e1003990. doi: 10.1371/journal.pgen.1003990 (PMC3854840; doi:10.1371/journal.pgen.1003990)

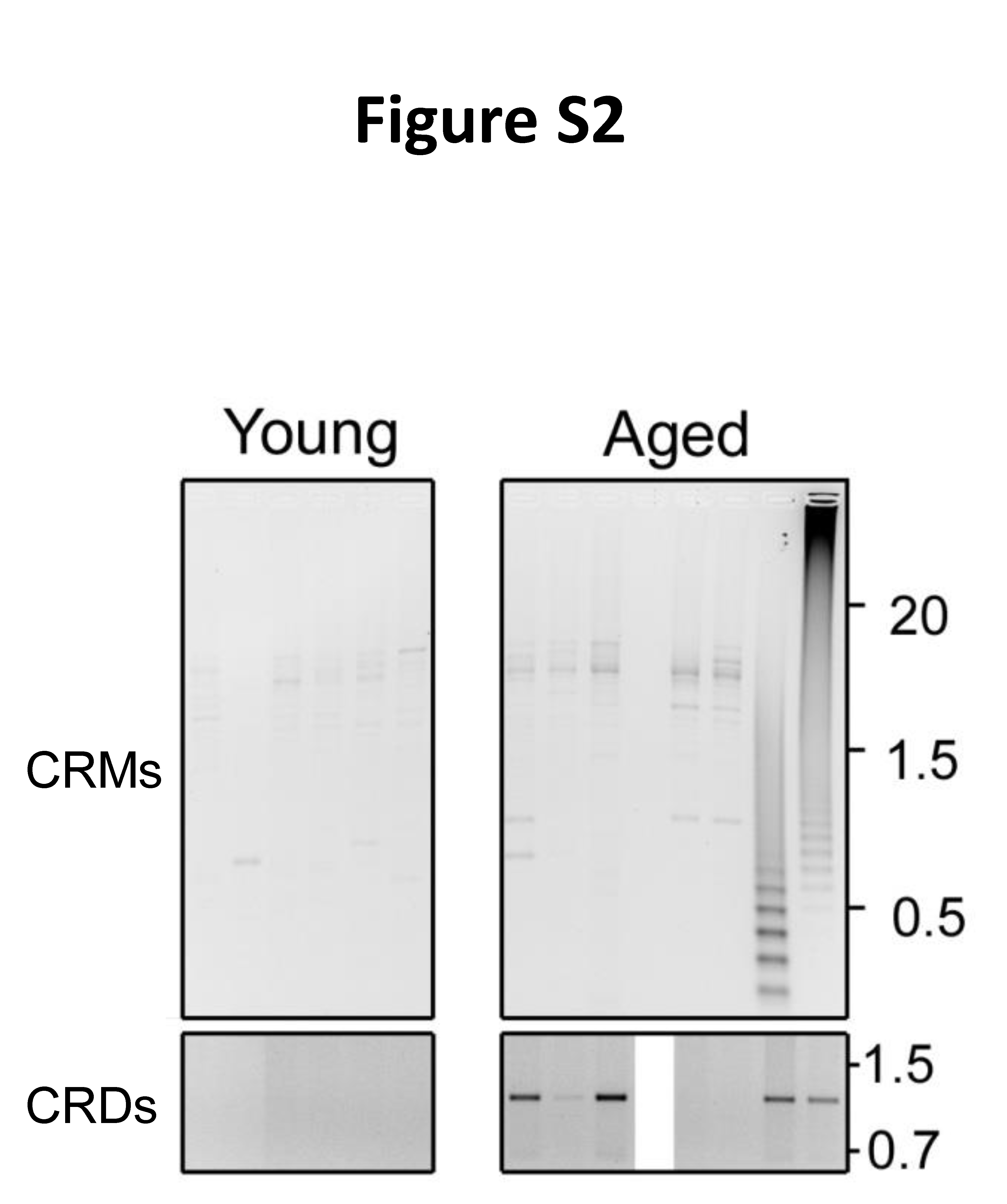

Supplement: Figure S2 — CRMs and CRDs detected in putamen are not universally present in cerebellum. Gel showing the lack of amplification of CRM products in cerebellum DNA of the cases under study using an identical PCR to that shown in Figure 1F. Sample order as in Figure 1F and Table S1. The two right-hand lanes in the panel of aged samples are positive controls: cerebellum from an additional case spiked with 1/50 dilution of A17 putamen mtDNA and a CRM positive putamen sample. Lower panel, PCR for CRDs using breakpoint-specific primer as in Figure 1F. Lane order as upper panel. No cerebellum specimen was available for A17 hence there is a blank space in the lower panel and empty lane directly above in upper panel. (TIF) [file pgen.1003990.s002.tif]

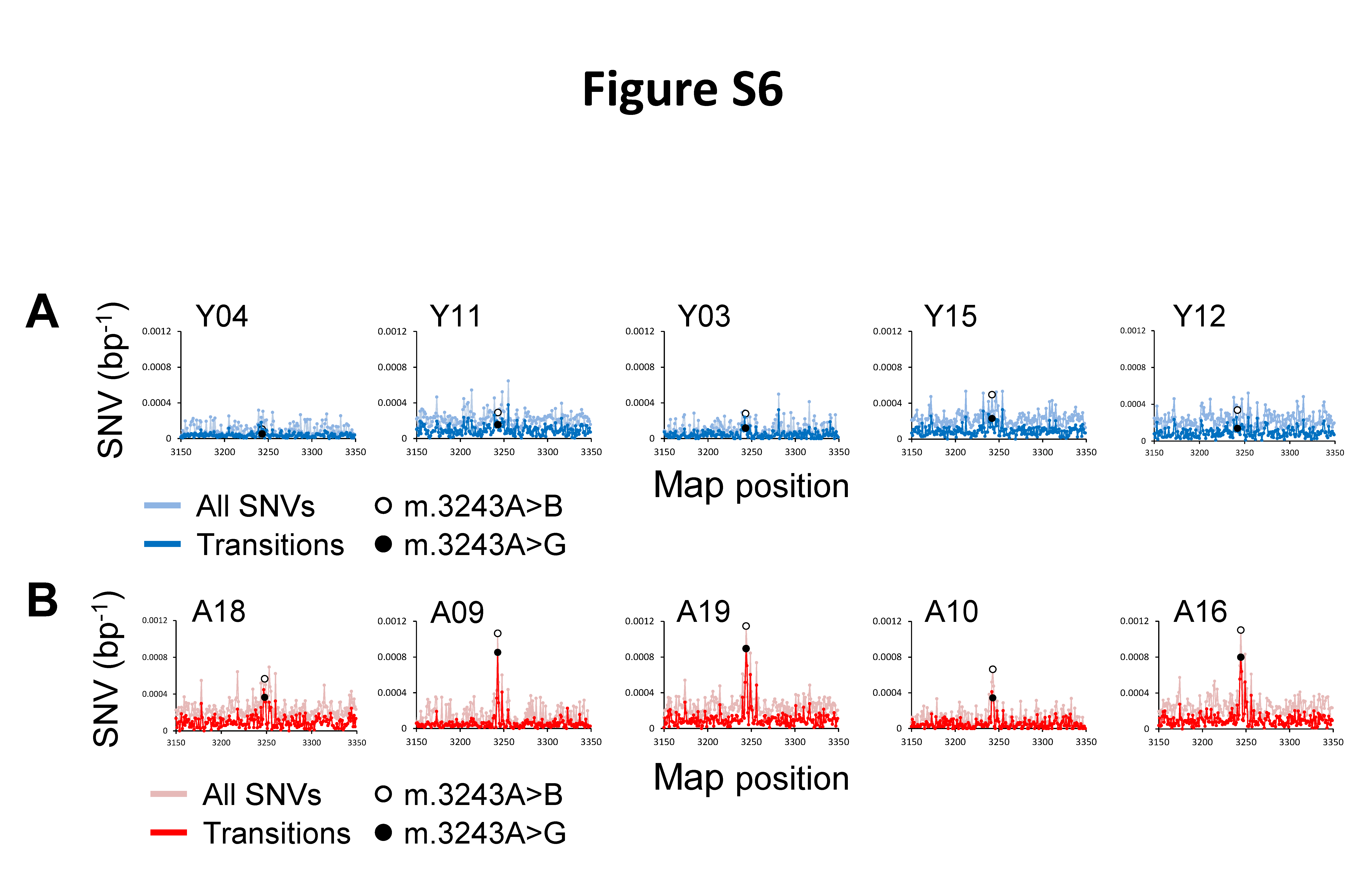

Supplement: Figure S6 — SNV clustering around m.3243 is observed in aged putamen. SNV frequencies with samples arranged left to right by increasing age, (A) young cohort, (B) aged cohort. (TIF) [file pgen.1003990.s006.tif]

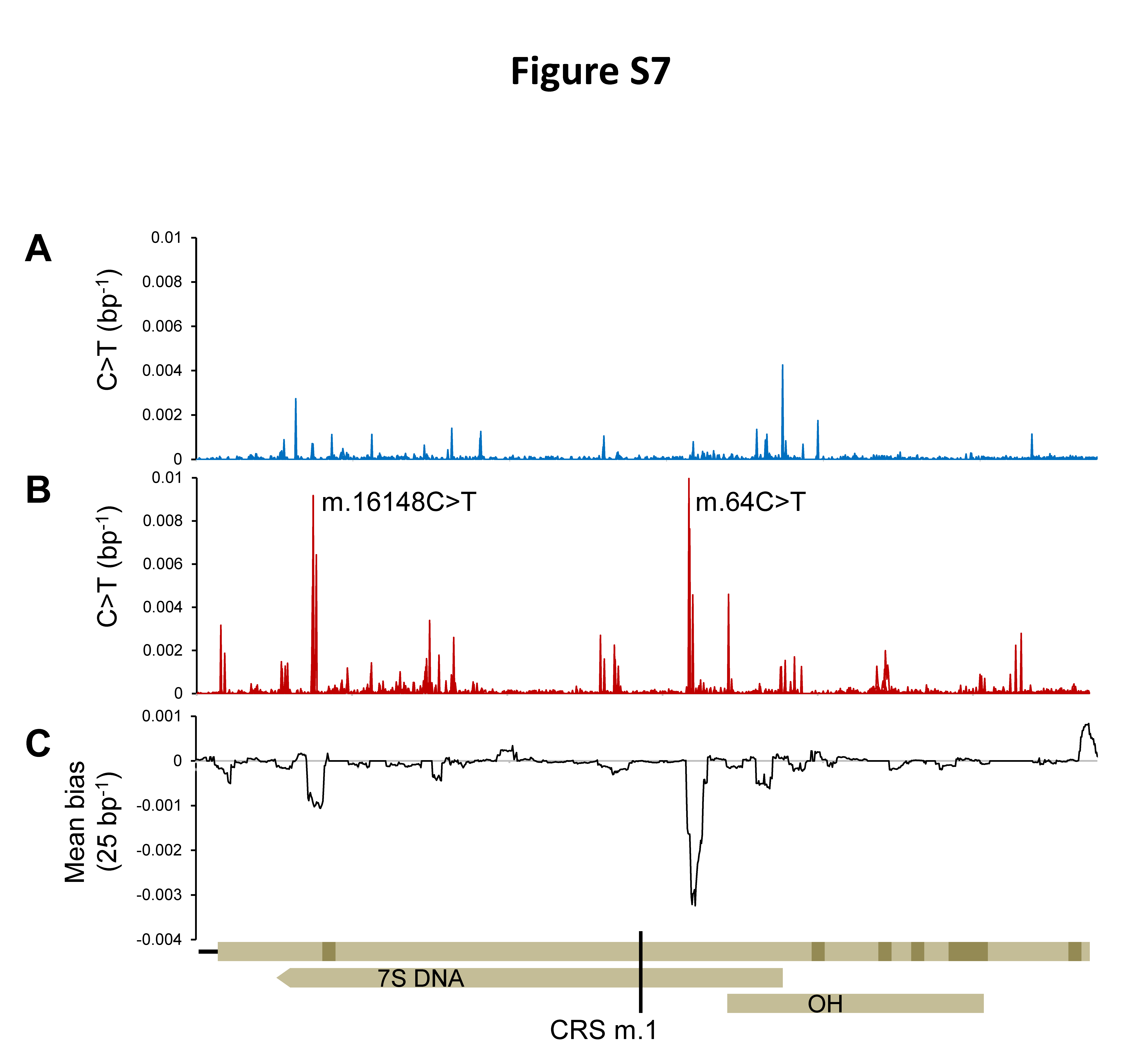

Supplement: Figure S7 — Overlaid C>T frequencies in for all young (A) and aged (B) samples. Peaks for m.64C>T and m.16148C>T indicated in (b). (C) Difference between mean [G>A]-[C>T] bias over 25 bp rolling average for young vs. aged samples aligned to above. Control region features indicated below each column as in Fig. 1C, top bar shows entire control region (light shading) with features indicated, left to right (dark shading): termination associated sequence, conserved sequence boxes I, II and III, Light-strand promoter and heavy strand promoter-1. Middle bar shows the 7S DNA with arrow indicating 3′ end. Lower bar defines heavy strand origin of replication (OH). CRS m.1, indicates first base of CRS numbering. (TIF) [file pgen.1003990.s007.tif]

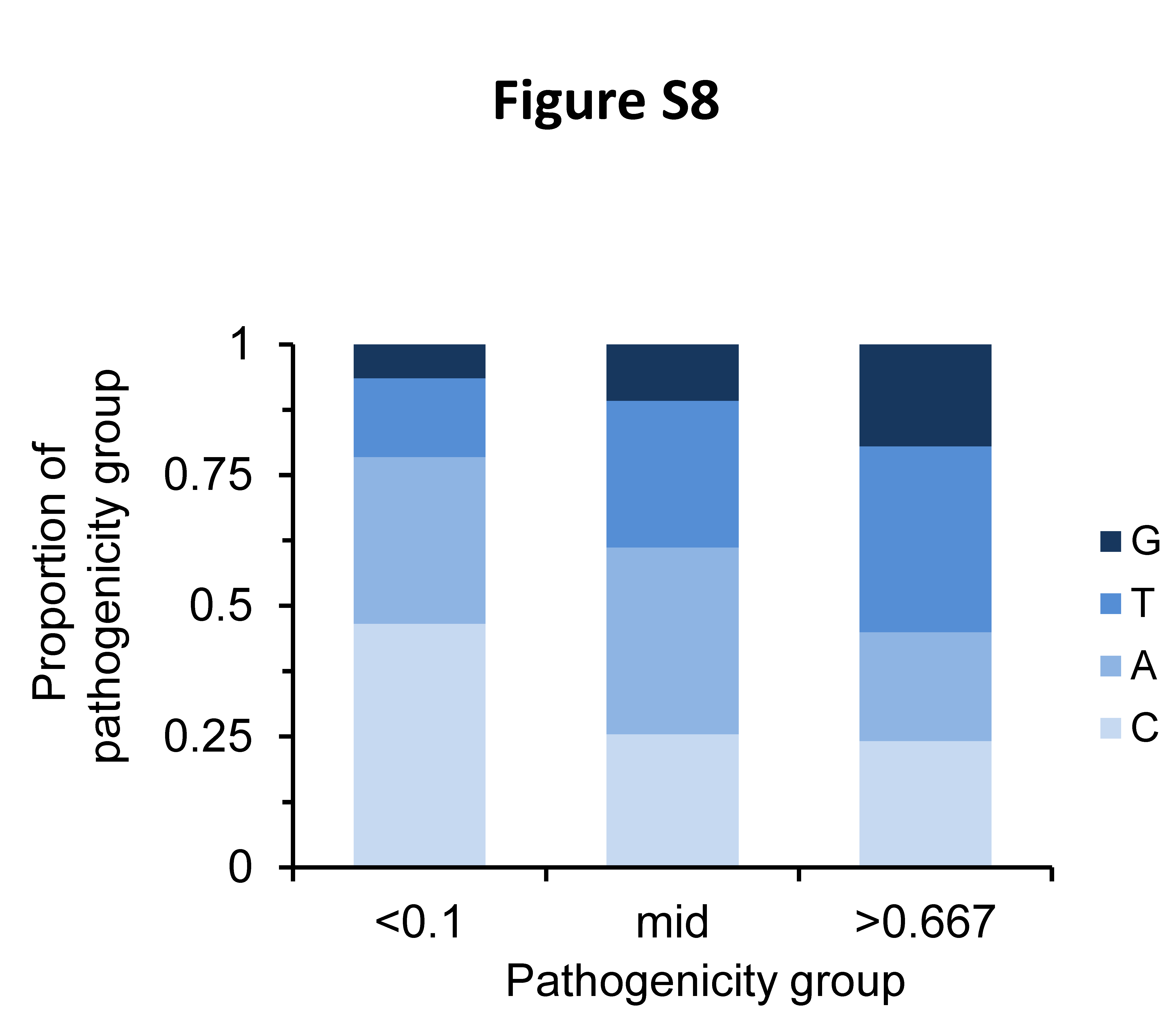

Supplement: Figure S8 — Bases counts as proportion of total bases in each pathogenicity group used for identifying pathogenicity skew. G>A and T>C mutations have the highest increase in frequency in the coding region and predominate in the group of transitions with the highest predicted pathogenicity. (TIF) [file pgen.1003990.s008.tif]

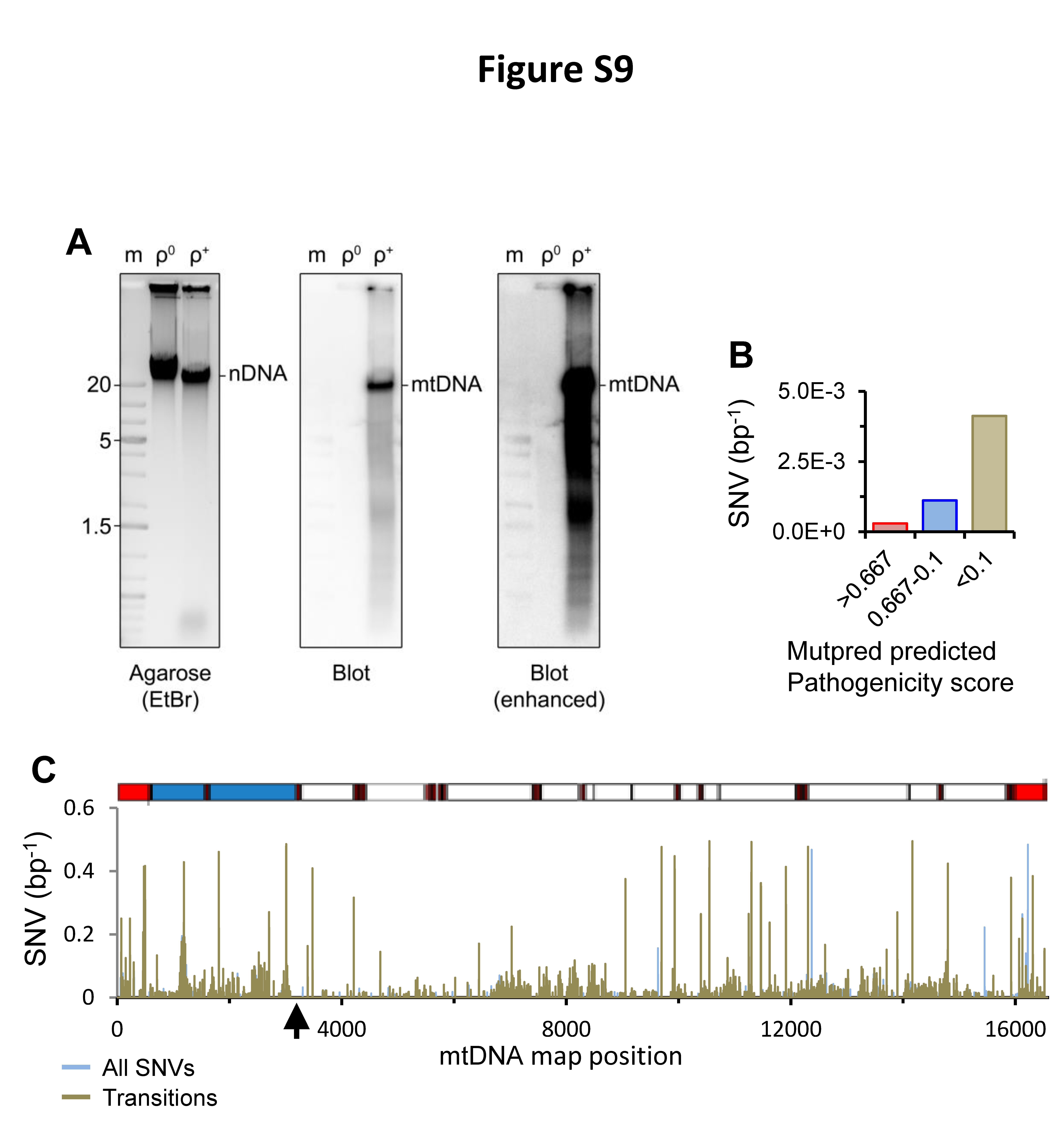

Supplement: Figure S9 — SNV analysis of mtDNA alignment of total DNA extracted from 143B.206 ρ0 cells lacking mtDNA. (A) Demonstration of the absence of mtDNA in the ρ0 cells, using the same DNA sample as used for library synthesis. Left-hand panel shows 0.7% agarose gel of 4.5 µg of XhoI-digested total DNA from ρ0 cells and wild-type 143B ρ+ cells which contain mtDNA, stained with ethidium bromide (EtBr). XhoI linearizes mtDNA by cutting once at m.14955. Middle panel is an image from a Cyclone phosphoimager (Perkin Elmer) of a blot of the gel in the left-hand hybridized with a probe against m.13385_15311. Note the absence of mtDNA signal in the ρ0 lane. Right-hand panel is the same image enhanced confirming the absence of signal. In each, m = molecular weight marker (Kb), ρ0 = 143B.206 ρ0 cell DNA, ρ+ = 143B ρ+ cell DNA. Attempts to quantify the region in the ρ0 lane equivalent to the mtDNA band in the ρ+ lane found relative signal to be 300–725-fold lower, below the quantification limit for Southern blotting. Furthermore, no evidence of any band equivalent mtDNA was present in the ρ0 lane. (B) Average frequencies of transition pseudo-SNVs called in the ρ0 alignment at bases with Mutpred scores of >0.667, 0.666-0.1 and <0.1. Note the distribution is the opposite of that seen in Mito-Seq assembles (Fig. 3C). (C) The distribution and frequency of SNVs called using identical detection parameters as the putamen samples. mtDNA map is given above depicting rRNA genes (blue), tRNA genes (black bars), protein coding genes (white) and the control region (red). Note lack of clustering in the control region and the absence of calls between m.3100–m.3300 (arrow), encompassing m.3243. (TIF) [file pgen.1003990.s009.tif]
