# Supplementary material for: Somatic mtDNA Mutation Spectra in the Aging Human Putamen
Source: PLoS Genet. 2013 Dec 5;9(12):e1003990. doi: 10.1371/journal.pgen.1003990 (PMC3854840; doi:10.1371/journal.pgen.1003990)
Supplement: Figure S5 — Reproducible differences in SNV frequencies between young and aged cohorts allowed normalization of SNV data from different sequencing runs. (A) Average frequencies of SNVs with a frequency of <0.01 bp−1 for each sample from the two sequencing runs that encompass all samples. Numbers above each plot cross-reference data in table (C) below. (B) Average frequencies of transitions with a frequency of <0.01 bp−1 for each sample. (C) Summary table of data relating to plots above. A-Y, mean frequency of aged cohort minus mean frequency of young cohort for each data set; ΔFrequency/yr, gradient of mean frequency vs. age. (D) Detail of SNV frequency vs. rank frequency (high-low) for SNVs with a frequency <0.01 for all samples. Samples A17 and Y13 (indicated) were considered outliers and were excluded from SNV analysis. (PDF) [file pgen.1003990.s005.pdf]

Figure S5

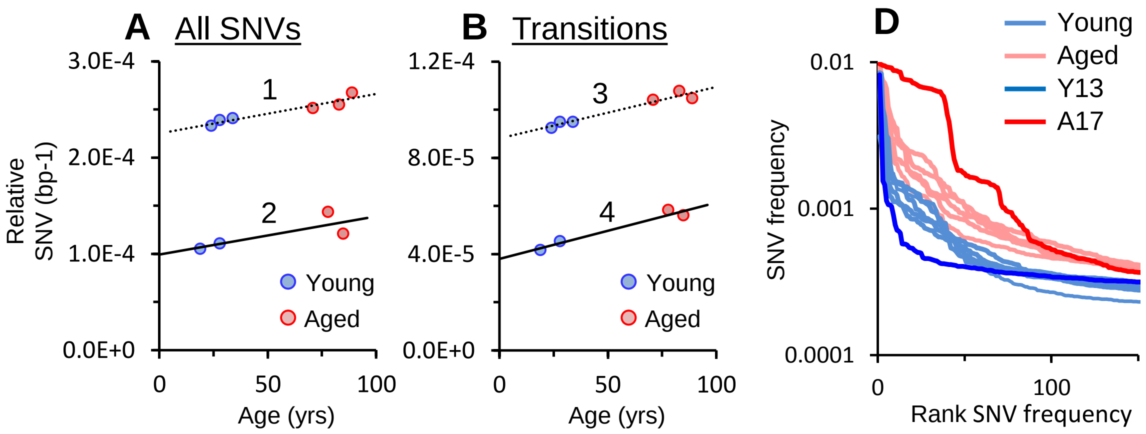

C

Plot	Data	A-Y	ΔFrequency/yr	R^2	Intercept	Run
1	All SNVs	2.03e-5	4.09e-7	0.91	2.25e-4	2
2	All SNVs	1.30e-5	3.95e-7	0.62	9.93e-5	1
3	Transitions	1.15e-5	2.14e-7	0.94	8.80e-5	2
4	Transitions	1.37e-5	2.34e-7	0.96	3.80e-5	1

Figure S5 Commentary.

Quality based SNV filtering, as implemented in this study, is a robust means of removing uncertain SNV calls caused by errors during sequencing runs. However, it cannot address the full spectrum of potential errors that occur throughout an NGS workflow and is incapable of removing errors that do not influence call quality, such as base incorporation errors during library preparation. The extent of NGS errors that cannot be removed using simple quality filtering was recently revealed using duplex consensus sequence filtering [27]. We determined that the high coverage and absence of significance filtering in our study would provide consistent un-filterable error rates across all samples. While this would reduce the confidence in the absolute mean SNV frequency of a given sample, the differences between samples within an NGS workflow would still be valid. Our data (Fig. S5) support this assumption given the reproducible A-Y values, the near parallel ΔFrequency/yr gradients and high R^2 values, yet differing intercept values for each sequencing run. In line with transitions having low false call rates [27] and being the most prevalent form of mtDNA variant, the data is stronger for transitions than for total SNVs (transitions plus transversions). In our view this allows for normalization the SNV data from the two sequencing runs and validates findings derived from A-Y values and ΔFrequency/yr gradients. All sequencing data was handled identically and bioinformatic variables can be ruled out as a basis for the difference between sequencing run SNV frequencies.
