# Supplementary material for: Somatic mtDNA Mutation Spectra in the Aging Human Putamen
Source: PLoS Genet. 2013 Dec 5;9(12):e1003990. doi: 10.1371/journal.pgen.1003990 (PMC3854840; doi:10.1371/journal.pgen.1003990)
Supplement: Table S1 — Summary of cases and alignment data. “% mtDNA reads” is the percentage of total sequencing reads that align to mtDNA. “SD as %” is the standard deviation in coverage as a percentage of average coverage. “Run” identifies which sequencing workflow each sample was part of. Input reads for ρ0 assembly was 47,991,218 and the mean for putamen libraries 46,881,984. (PDF) [file pgen.1003990.s010.pdf]

Table S1. Summary of cases and alignment data.

ID	Age (yrs)	Cause of death	mtDNA Haplotype	% mtDNA reads	Average coverage	SD as %	Run
Y03	28	Fibromuscaular dysplasia of the sinoatrial nodal artery	J1c2	44.9	76,199	18.9	1
Y04	19	Gunshot wound	U2e2a1	60.1	172,949	19.0	1
Y11	24	Motor vehicle accident	H66a	49.0	218,353	n/a	2
Y12	34	Acute asthmatic bronchitis	A2ac	51.4	141,321	13.7	2
Y13	29	Electrocution	K1a1b1a	49.0	108,648	14.3	2
Y15	28	Motor vehicle accident	A2	62.3	167,205	14.3	2
A09	78	Cardiopulmonary collapse	K1a1b1e	29.1	79,988	24.3	1
A10	85	Ischemic cardiomyopathy, coronary artery disease	H1as	12.0	47,824	22.5	1
A16	89	pneumonia and stroke	V16	44.6	106,134	14.0	2
A17	67	Metastatic lung carcinoma	T1a1	51.6	153,349	13.5	2
A18	71	Myocardial infarction	W6a	47.5	112,206	13.9	2
A19	83	Renal and lung failure	J1c1a	43.6	134,278	13.0	2
ρ^0	13	Osteosarcoma, cell line	(R, U8b)	0.03	91	24.2	1

"% mtDNA reads" is the percentage of total sequencing reads that align to mtDNA. "SD as %" is the standard deviation in coverage as a percentage of average coverage. "Run" identifies which sequencing workflow each sample was part of. Input reads for ρ^0 assembly was 47,991,218 and the mean for putamen libraries 46,881,984.
